# Poly[μ-aqua-μ-(*N*,4-di­chloro-2-methyl­benzene­sulfonamidato)-potassium]

**DOI:** 10.1107/S1600536813015845

**Published:** 2013-07-03

**Authors:** H. S. Spandana, Sabine Foro, B. Thimme Gowda

**Affiliations:** a Department of Chemistry, Mangalore University, Mangalagangotri 574 199, Mangalore, India; b Institute of Materials Science, Darmstadt University of Technology, Petersenstrasse 23, D-64287 Darmstadt, Germany; c Jnanabharathi Campus, Bangalore University, Bangalore 560 056, India

## Abstract

In the title compound, [K(C_7_H_6_Cl_2_NO_2_S)(H_2_O)]_*n*_, the K^+^ cation is hepta­coordinated by two water O atoms, a sulfonyl O atom from each of four different *N*,4-dichloro-2-methyl­benzene­sulfonamidate anions and a Cl atom of one of the anions. Further, K—O—K bridges form extensive polymeric chains along the *b* axis. In the crystal structure, the anions are linked into layers parallel to (100) by O—H⋯Cl and O—H⋯N hydrogen bonds.

## Related literature

For preparation of *N*-halo­aryl­sulfonamides, see: Gowda & Mahadevappa (1983 ▶). For studies of the effect of substituents on the structures of *N*-halo­aryl­sulfonamidates, see: George *et al.* (2000 ▶); Gowda *et al.* (2007 ▶, 2011*a* ▶,*b* ▶,*c* ▶); Olmstead & Power (1986 ▶). For restrained geometry, see: Nardelli (1999 ▶). 
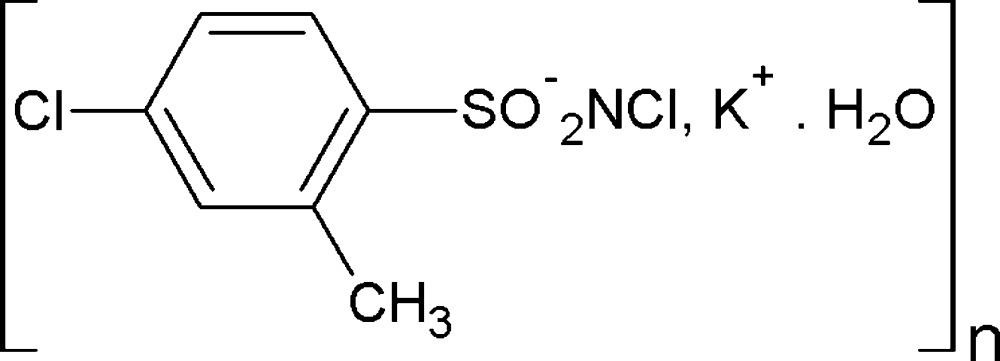


## Experimental

### 

#### Crystal data

[K(C_7_H_6_Cl_2_NO_2_S)(H_2_O)]
*M* *_r_* = 296.20
Monoclinic, 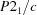

*a* = 15.190 (1) Å
*b* = 11.3138 (9) Å
*c* = 6.7200 (5) Å
β = 100.627 (7)°
*V* = 1135.07 (14) Å^3^
*Z* = 4
Mo *K*α radiation
μ = 1.11 mm^−1^
*T* = 293 K
0.44 × 0.28 × 0.06 mm

#### Data collection

Oxford Diffraction Xcalibur diffractometer with a Sapphire CCD detector
Absorption correction: multi-scan (*CrysAlis RED*; Oxford Diffraction, 2009 ▶) *T*_min_ = 0.642, *T*_max_ = 0.937
4588 measured reflections
2297 independent reflections
2043 reflections with *I* > 2σ(*I*)
*R*_int_ = 0.022

#### Refinement

*R*[*F* ^2^ > 2σ(*F* ^2^)] = 0.076
*wR*(*F* ^2^) = 0.193
*S* = 1.28
2297 reflections
144 parameters
3 restraints
H atoms treated by a mixture of independent and constrained refinement
Δρ_max_ = 0.96 e Å^−3^
Δρ_min_ = −0.45 e Å^−3^

### 

Data collection: *CrysAlis CCD* (Oxford Diffraction, 2009 ▶); cell refinement: *CrysAlis RED* (Oxford Diffraction, 2009 ▶); data reduction: *CrysAlis RED*; program(s) used to solve structure: *SHELXS97* (Sheldrick, 2008 ▶); program(s) used to refine structure: *SHELXL97* (Sheldrick, 2008 ▶); molecular graphics: *PLATON* (Spek, 2009 ▶); software used to prepare material for publication: *SHELXL97*.

## Supplementary Material

Crystal structure: contains datablock(s) I, global. DOI: 10.1107/S1600536813015845/sj5326sup1.cif

Structure factors: contains datablock(s) I. DOI: 10.1107/S1600536813015845/sj5326Isup2.hkl

Supplementary material file. DOI: 10.1107/S1600536813015845/sj5326Isup3.cml

Additional supplementary materials:  crystallographic information; 3D view; checkCIF report

## Figures and Tables

**Table 1:** Hydrogen-bond geometry (Å, °)

*D*—H⋯*A*	*D*—H	H⋯*A*	*D*⋯*A*	*D*—H⋯*A*
O3—H31⋯N1^i^	0.85 (2)	2.06 (2)	2.901 (9)	173 (9)
O3—H32⋯Cl1^ii^	0.85 (2)	2.86 (5)	3.603 (6)	148 (9)

Symmetry codes: (i) 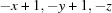; (ii) 
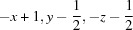
.

**Fig. 1.**
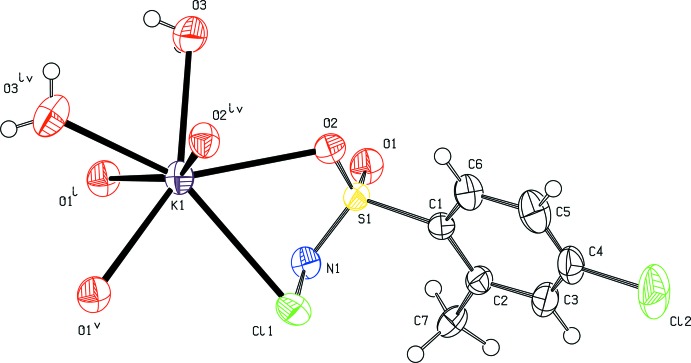
Molecular structure of the title compound, showing the atom labelling scheme for the asymmetric unit and extended to show the coordination geometry about K+. The displacement ellipsoids are drawn at the 50% probability level. The H atoms are represented as small spheres of arbitrary radii.

**Fig. 2.**
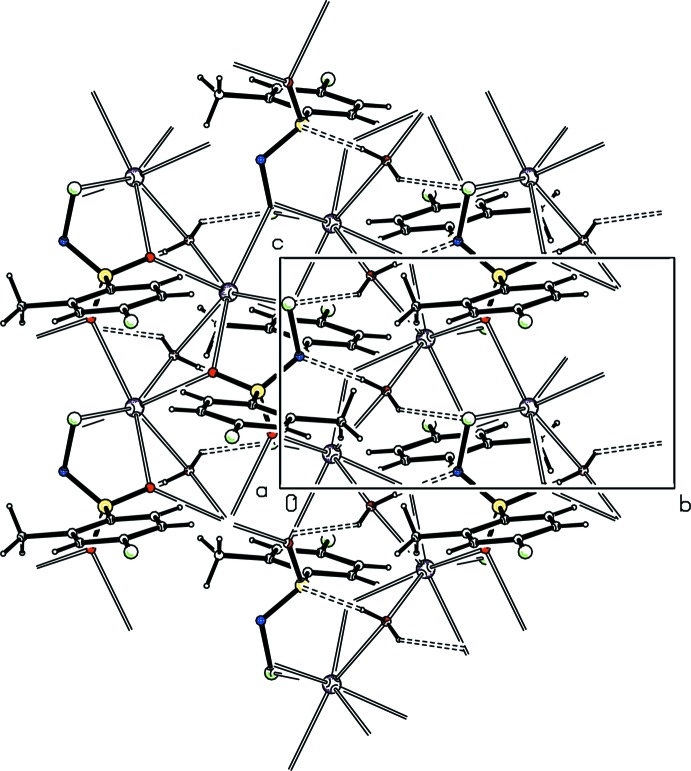
Molecular packing of the title compound with hydrogen bonding shown as dashed lines.

**Fig. 3.**
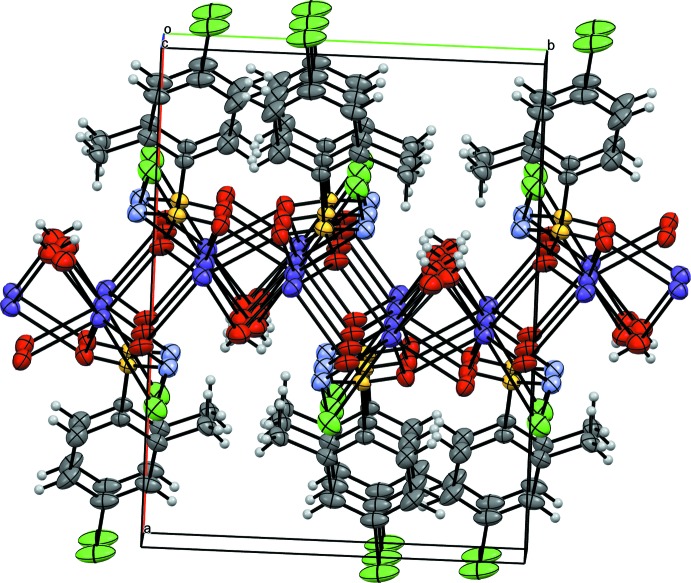
Coordination polymer generated by K–O–K bridges which form extensive polymer chains along the b axis,

## supplementary crystallographic information

### Comment

The present work was undertaken in order to explore the effect of replacing sodium ion by potassium ion on the solid state structures of metal salts of *N*-haloarylsulfonamidates(Gowda *et al.*, 2007, 2011**a*,*b*,c*), the structure of potassium *N*-chloro-2-methyl-4-chlorobenzene- sulfonamidate monohydrate (I) has been determined (Fig. 1). The structure of (I) resembles those of potassium *N*-chloro-benzenesulfonamidate monohydrate (II) (Gowda *et al.*, 2007), potassium *N*-chloro-4-chlorobenzenesulfonamidate monohydrate (III) (Gowda *et al.*, 2011*b*), potassium *N*-chloro-2-methyl-benzenesulfonamidate monohydrate (IV) (Gowda *et al.*, 2011*c*) and other sodium *N*-chloroarylsulfonamidates (George *et al.*, 2000; Olmstead & Power, 1986).

In the title compound, K^+^ ion is hepta coordinated by two O atoms from two different water molecules, sulfonyl O atoms of four different *N*-chloro-2-methyl-4-chlorobenzenesulfonamide anions and the Cl atom of the N—Cl bond in one of the *N*-chloro-2-methyl-4-chlorobenzene- sulfonamidate anions, similar to the coordination observed in II, III and IV. However, this is in contrast to the situation for potassium *N*-chloro-2-chlorobenzenesulfonamidate sesquihydrate (Gowda *et al.*, 2011*a*) where the K^+^ cation acheives hepta coordination by binding three O atoms from three different water molecules and four sulfonyl O atoms of three different *N*-chloro-2-chlorobenzenesulfonamidate anions.

The S—N distance of 1.580 (6) Å is consistent with a S—N double bond and is in agreement with the observed values of 1.581 (4) Å in (II), 1.588 (2) Å in (III) and 1.584 (3) Å in (IV).

In the crystal structure the anions are linked by intermolecular O3—H32···Cl1 and O3—H31···N1 hydrogen bonding into layers (Fig. 2 and Table 1). Further, K– O –K bridges form extensive polymer chains along the *b* axis, generating a coordination polymer (Fig. 3).

### Experimental

The title compound was prepared by a method similar to the one described by Gowda & Mahadevappa (Gowda & Mahadevappa, 1983). 2 g of 2-methyl-4-chlorobenzenesulfonamide was dissolved with stirring in 40 ml of 5*M* KOH at 70° C. Pure chlorine gas was bubbled through clear aqueous solution for about 1 hr. The precipitated potassium salt of *N*-chloro-2-methyl-4-chlorobenzenesulfonamidate was filtered under suction, washed quickly with a minimum quantity of ice cold water. The purity of the compound was checked by determining its melting point (170 ° C) and estimating, iodometrically, the amount of active chlorine present in it. It was further characterized from its infrared spectrum.

Plate like colourless single crystals of the title compound used in the X-ray diffraction studies were obtained from its aqueous solution at room temperature.

### Refinement

The O-bound H atoms were located in difference map and were refined with restrained geometry (Nardelli, 1999), *viz*. O—H distances were restrained to 0.85 (2) Å and H—H distance was restrained to 1.365 Å, thus leading to the angle of 107°.

H atoms bonded to C were positioned with idealized geometry using a riding model with the aromatic C—H = 0.93 Å, methyl C—H = 0.96 Å. All H atoms were refined with isotropic displacement parameters set at 1.2 *U*_eq_(C-aromatic, N, O) and 1.5 *U*_eq_(*C*-methyl) of the parent atom.

The (6 1 1, -1 0 6, -10 1 1) reflections had a poor disagreement with their calculated values and were omitted from the refinement.

The crystal was refined with the twin law (1 0 0.835/0 - 1 0/0 0 - 1).

### Crystal data

**Table:**

[K(C_7_H_6_Cl_2_NO_2_S)(H_2_O)]	*F*(000) = 600
*M**_r_* = 296.20	*D*_x_ = 1.733 Mg m^−^^3^
Monoclinic, *P*2_1_/*c*	Mo *K*α radiation, λ = 0.71073 Å
Hall symbol: -P 2ybc	Cell parameters from 2383 reflections
*a* = 15.190 (1) Å	θ = 3.1–27.8°
*b* = 11.3138 (9) Å	µ = 1.11 mm^−^^1^
*c* = 6.7200 (5) Å	*T* = 293 K
β = 100.627 (7)°	Plate, colourless
*V* = 1135.07 (14) Å^3^	0.44 × 0.28 × 0.06 mm
*Z* = 4	

### Data collection

**Table:**

Oxford Diffraction Xcalibur diffractometer with a Sapphire CCD detector	2297 independent reflections
Radiation source: fine-focus sealed tube	2043 reflections with *I* > 2σ(*I*)
Graphite monochromator	*R*_int_ = 0.022
Rotation method data acquisition using ω scans	θ_max_ = 26.4°, θ_min_ = 3.1°
Absorption correction: multi-scan (*CrysAlis RED*; Oxford Diffraction, 2009)	*h* = −18→16
*T*_min_ = 0.642, *T*_max_ = 0.937	*k* = −14→6
4588 measured reflections	*l* = −3→8

### Refinement

**Table:**

Refinement on *F*^2^	Primary atom site location: structure-invariant direct methods
Least-squares matrix: full	Secondary atom site location: difference Fourier map
*R*[*F*^2^ > 2σ(*F*^2^)] = 0.076	Hydrogen site location: inferred from neighbouring sites
*wR*(*F*^2^) = 0.193	H atoms treated by a mixture of independent and constrained refinement
*S* = 1.28	*w* = 1/[σ^2^(*F*_o_^2^) + (0.0345*P*)^2^ + 9.1024*P*] where *P* = (*F*_o_^2^ + 2*F*_c_^2^)/3
2297 reflections	(Δ/σ)_max_ = 0.002
144 parameters	Δρ_max_ = 0.96 e Å^−^^3^
3 restraints	Δρ_min_ = −0.45 e Å^−^^3^

### Special details

**Table:**

Geometry. All e.s.d.'s (except the e.s.d. in the dihedral angle between two l.s. planes) are estimated using the full covariance matrix. The cell e.s.d.'s are taken into account individually in the estimation of e.s.d.'s in distances, angles and torsion angles; correlations between e.s.d.'s in cell parameters are only used when they are defined by crystal symmetry. An approximate (isotropic) treatment of cell e.s.d.'s is used for estimating e.s.d.'s involving l.s. planes.
Refinement. Refinement of *F*^2^ against ALL reflections. The weighted *R*-factor *wR* and goodness of fit *S* are based on *F*^2^, conventional *R*-factors *R* are based on *F*, with *F* set to zero for negative *F*^2^. The threshold expression of *F*^2^ > σ(*F*^2^) is used only for calculating *R*-factors(gt) *etc*. and is not relevant to the choice of reflections for refinement. *R*-factors based on *F*^2^ are statistically about twice as large as those based on *F*, and *R*- factors based on ALL data will be even larger.

### Fractional atomic coordinates and isotropic or equivalent isotropic displacement parameters (Å^2^)

**Table:**

	*x*	*y*	*z*	*U*_iso_*/*U*_eq_	
C1	0.2356 (4)	0.4297 (6)	0.1358 (9)	0.0268 (13)	
C2	0.1902 (4)	0.5272 (6)	0.1947 (9)	0.0303 (13)	
C3	0.1045 (5)	0.5082 (8)	0.2363 (10)	0.0404 (17)	
H3	0.0720	0.5711	0.2749	0.049*	
C4	0.0680 (5)	0.3960 (8)	0.2203 (11)	0.0441 (18)	
C5	0.1126 (5)	0.3003 (8)	0.1652 (12)	0.0482 (19)	
H5	0.0870	0.2254	0.1570	0.058*	
C6	0.1975 (5)	0.3183 (7)	0.1216 (10)	0.0378 (15)	
H6	0.2291	0.2547	0.0824	0.045*	
C7	0.2269 (5)	0.6544 (6)	0.2104 (11)	0.0364 (15)	
H7A	0.2240	0.6863	0.0769	0.055*	
H7B	0.2880	0.6539	0.2803	0.055*	
H7C	0.1916	0.7024	0.2837	0.055*	
N1	0.3485 (4)	0.5488 (5)	−0.0738 (9)	0.0372 (13)	
O1	0.4057 (3)	0.4798 (5)	0.2621 (7)	0.0413 (12)	
O2	0.3645 (3)	0.3298 (4)	−0.0029 (7)	0.0388 (11)	
O3	0.5671 (4)	0.2308 (6)	−0.0773 (10)	0.0545 (15)	
H31	0.595 (5)	0.292 (5)	−0.027 (13)	0.065*	
H32	0.598 (5)	0.197 (7)	−0.153 (12)	0.065*	
K1	0.44984 (10)	0.36919 (13)	−0.3505 (2)	0.0356 (4)	
Cl1	0.27213 (14)	0.52161 (18)	−0.3006 (3)	0.0476 (5)	
Cl2	−0.03915 (14)	0.3782 (3)	0.2755 (4)	0.0797 (9)	
S1	0.34542 (10)	0.44325 (14)	0.0788 (2)	0.0283 (4)	

### Atomic displacement parameters (Å^2^)

**Table:**

	*U*^11^	*U*^22^	*U*^33^	*U*^12^	*U*^13^	*U*^23^
C1	0.023 (3)	0.033 (3)	0.023 (3)	0.000 (2)	0.002 (2)	0.002 (2)
C2	0.029 (3)	0.038 (4)	0.024 (3)	0.005 (3)	0.005 (2)	0.000 (3)
C3	0.029 (3)	0.064 (5)	0.030 (3)	0.006 (3)	0.009 (3)	0.002 (3)
C4	0.027 (3)	0.074 (6)	0.032 (4)	−0.005 (4)	0.007 (3)	0.009 (4)
C5	0.048 (4)	0.051 (5)	0.047 (4)	−0.023 (4)	0.013 (3)	−0.001 (4)
C6	0.041 (4)	0.042 (4)	0.032 (3)	−0.006 (3)	0.011 (3)	−0.003 (3)
C7	0.034 (3)	0.037 (4)	0.041 (4)	0.008 (3)	0.012 (3)	−0.008 (3)
N1	0.036 (3)	0.038 (3)	0.039 (3)	−0.001 (2)	0.011 (2)	0.004 (3)
O1	0.028 (2)	0.056 (3)	0.038 (3)	−0.006 (2)	−0.001 (2)	−0.002 (2)
O2	0.042 (3)	0.033 (3)	0.043 (3)	0.007 (2)	0.014 (2)	0.003 (2)
O3	0.040 (3)	0.056 (4)	0.071 (4)	0.001 (3)	0.018 (3)	0.013 (3)
K1	0.0345 (8)	0.0377 (8)	0.0359 (8)	−0.0039 (6)	0.0101 (6)	−0.0011 (6)
Cl1	0.0538 (11)	0.0549 (12)	0.0336 (9)	0.0095 (9)	0.0067 (8)	0.0111 (8)
Cl2	0.0338 (10)	0.143 (3)	0.0643 (15)	−0.0190 (13)	0.0154 (10)	0.0122 (16)
S1	0.0245 (7)	0.0323 (8)	0.0287 (8)	0.0019 (6)	0.0061 (6)	0.0014 (6)

### Geometric parameters (Å, º)

**Table:**

C1—C6	1.383 (9)	O1—S1	1.452 (5)
C1—C2	1.397 (9)	O1—K1^i^	2.758 (5)
C1—S1	1.786 (6)	O1—K1^ii^	2.855 (5)
C2—C3	1.397 (9)	O2—S1	1.446 (5)
C2—C7	1.540 (10)	O2—K1^iii^	2.703 (5)
C3—C4	1.381 (12)	O2—K1	2.907 (5)
C3—H3	0.9300	O3—K1^iii^	2.789 (6)
C4—C5	1.364 (12)	O3—K1	2.790 (6)
C4—Cl2	1.747 (7)	O3—H31	0.85 (2)
C5—C6	1.389 (10)	O3—H32	0.85 (2)
C5—H5	0.9300	K1—O2^iv^	2.703 (5)
C6—H6	0.9300	K1—O1^i^	2.758 (5)
C7—H7A	0.9600	K1—O3^iv^	2.789 (6)
C7—H7B	0.9600	K1—O1^v^	2.855 (5)
C7—H7C	0.9600	K1—Cl1	3.272 (2)
N1—S1	1.580 (6)	K1—H31	2.93 (9)
N1—Cl1	1.763 (6)	K1—H32	3.09 (9)
N1—K1	3.319 (6)		
			
C6—C1—C2	121.2 (6)	O1^i^—K1—O1^v^	88.21 (14)
C6—C1—S1	117.4 (5)	O3^iv^—K1—O1^v^	75.28 (18)
C2—C1—S1	121.5 (5)	O3—K1—O1^v^	149.33 (17)
C1—C2—C3	117.5 (6)	O2^iv^—K1—O2	85.58 (13)
C1—C2—C7	124.5 (6)	O1^i^—K1—O2	112.15 (15)
C3—C2—C7	118.0 (6)	O3^iv^—K1—O2	143.26 (18)
C4—C3—C2	120.1 (7)	O3—K1—O2	73.18 (16)
C4—C3—H3	120.0	O1^v^—K1—O2	137.32 (15)
C2—C3—H3	120.0	O2^iv^—K1—Cl1	97.50 (12)
C5—C4—C3	122.6 (6)	O1^i^—K1—Cl1	106.88 (13)
C5—C4—Cl2	119.6 (6)	O3^iv^—K1—Cl1	153.12 (15)
C3—C4—Cl2	117.8 (6)	O3—K1—Cl1	131.81 (14)
C4—C5—C6	117.8 (7)	O1^v^—K1—Cl1	78.62 (11)
C4—C5—H5	121.1	O2—K1—Cl1	59.99 (10)
C6—C5—H5	121.1	O2^iv^—K1—N1	118.84 (15)
C1—C6—C5	120.8 (7)	O1^i^—K1—N1	86.17 (15)
C1—C6—H6	119.6	O3^iv^—K1—N1	164.53 (17)
C5—C6—H6	119.6	O3—K1—N1	106.30 (18)
C2—C7—H7A	109.5	O1^v^—K1—N1	100.78 (15)
C2—C7—H7B	109.5	O2—K1—N1	47.31 (14)
H7A—C7—H7B	109.5	Cl1—K1—N1	31.03 (11)
C2—C7—H7C	109.5	O2^iv^—K1—H31	105.9 (7)
H7A—C7—H7C	109.5	O1^i^—K1—H31	64.2 (10)
H7B—C7—H7C	109.5	O3^iv^—K1—H31	79.3 (15)
S1—N1—Cl1	109.6 (3)	O3—K1—H31	16.8 (7)
S1—N1—K1	88.5 (2)	O1^v^—K1—H31	145.6 (15)
Cl1—N1—K1	73.0 (2)	O2—K1—H31	75.3 (15)
S1—O1—K1^i^	135.3 (3)	Cl1—K1—H31	127.2 (14)
S1—O1—K1^ii^	130.8 (3)	N1—K1—H31	97.6 (13)
K1^i^—O1—K1^ii^	91.79 (14)	O2^iv^—K1—H32	84.2 (14)
S1—O2—K1^iii^	135.3 (3)	O1^i^—K1—H32	79.0 (14)
S1—O2—K1	108.6 (2)	O3^iv^—K1—H32	59.3 (10)
K1^iii^—O2—K1	100.27 (15)	O3—K1—H32	15.5 (9)
K1^iii^—O3—K1	101.11 (18)	O1^v^—K1—H32	134.3 (10)
K1^iii^—O3—H31	116 (6)	O2—K1—H32	87.5 (11)
K1—O3—H31	91 (7)	Cl1—K1—H32	147.0 (10)
K1^iii^—O3—H32	129 (6)	N1—K1—H32	121.5 (9)
K1—O3—H32	103 (7)	H31—K1—H32	26.1 (7)
H31—O3—H32	108 (3)	N1—Cl1—K1	75.9 (2)
O2^iv^—K1—O1^i^	154.73 (16)	O2—S1—O1	115.8 (3)
O2^iv^—K1—O3^iv^	76.41 (16)	O2—S1—N1	113.1 (3)
O1^i^—K1—O3^iv^	78.81 (17)	O1—S1—N1	104.2 (3)
O2^iv^—K1—O3	89.17 (18)	O2—S1—C1	105.4 (3)
O1^i^—K1—O3	79.62 (17)	O1—S1—C1	108.1 (3)
O3^iv^—K1—O3	74.78 (11)	N1—S1—C1	110.1 (3)
O2^iv^—K1—O1^v^	90.22 (15)		
			
C6—C1—C2—C3	−0.7 (9)	Cl1—N1—K1—O3^iv^	118.7 (7)
S1—C1—C2—C3	−180.0 (5)	S1—N1—K1—O3	−38.4 (3)
C6—C1—C2—C7	−178.8 (6)	Cl1—N1—K1—O3	−149.5 (2)
S1—C1—C2—C7	2.0 (9)	S1—N1—K1—O1^v^	156.1 (2)
C1—C2—C3—C4	0.5 (10)	Cl1—N1—K1—O1^v^	45.1 (2)
C7—C2—C3—C4	178.7 (6)	S1—N1—K1—O2	8.75 (19)
C2—C3—C4—C5	0.3 (11)	Cl1—N1—K1—O2	−102.3 (3)
C2—C3—C4—Cl2	179.7 (5)	S1—N1—K1—Cl1	111.1 (3)
C3—C4—C5—C6	−0.8 (12)	S1—N1—Cl1—K1	−82.0 (3)
Cl2—C4—C5—C6	179.7 (6)	O2^iv^—K1—Cl1—N1	136.5 (2)
C2—C1—C6—C5	0.1 (10)	O1^i^—K1—Cl1—N1	−50.2 (2)
S1—C1—C6—C5	179.5 (6)	O3^iv^—K1—Cl1—N1	−148.9 (4)
C4—C5—C6—C1	0.6 (11)	O3—K1—Cl1—N1	40.8 (3)
K1^iii^—O3—K1—O2^iv^	−63.5 (2)	O1^v^—K1—Cl1—N1	−134.8 (2)
K1^iii^—O3—K1—O1^i^	139.3 (2)	O2—K1—Cl1—N1	56.0 (2)
K1^iii^—O3—K1—O3^iv^	−139.6 (3)	K1^iii^—O2—S1—O1	24.3 (5)
K1^iii^—O3—K1—O1^v^	−152.5 (3)	K1—O2—S1—O1	−103.1 (3)
K1^iii^—O3—K1—O2	22.15 (17)	K1^iii^—O2—S1—N1	144.5 (4)
K1^iii^—O3—K1—Cl1	35.9 (3)	K1—O2—S1—N1	17.1 (4)
K1^iii^—O3—K1—N1	56.4 (2)	K1^iii^—O2—S1—C1	−95.1 (4)
S1—O2—K1—O2^iv^	−146.95 (18)	K1—O2—S1—C1	137.5 (2)
K1^iii^—O2—K1—O2^iv^	67.6 (2)	K1^i^—O1—S1—O2	100.4 (4)
S1—O2—K1—O1^i^	51.6 (3)	K1^ii^—O1—S1—O2	−58.3 (4)
K1^iii^—O2—K1—O1^i^	−93.77 (18)	K1^i^—O1—S1—N1	−24.5 (5)
S1—O2—K1—O3^iv^	152.9 (3)	K1^ii^—O1—S1—N1	176.8 (3)
K1^iii^—O2—K1—O3^iv^	7.5 (4)	K1^i^—O1—S1—C1	−141.7 (4)
S1—O2—K1—O3	122.6 (3)	K1^ii^—O1—S1—C1	59.6 (4)
K1^iii^—O2—K1—O3	−22.82 (18)	Cl1—N1—S1—O2	57.2 (4)
S1—O2—K1—O1^v^	−61.4 (4)	K1—N1—S1—O2	−14.1 (3)
K1^iii^—O2—K1—O1^v^	153.17 (18)	Cl1—N1—S1—O1	−176.1 (3)
S1—O2—K1—Cl1	−45.7 (2)	K1—N1—S1—O1	112.5 (2)
K1^iii^—O2—K1—Cl1	168.94 (19)	Cl1—N1—S1—C1	−60.4 (4)
S1—O2—K1—N1	−10.1 (2)	K1—N1—S1—C1	−131.8 (2)
K1^iii^—O2—K1—N1	−155.5 (3)	C6—C1—S1—O2	8.7 (6)
S1—N1—K1—O2^iv^	59.9 (3)	C2—C1—S1—O2	−172.0 (5)
Cl1—N1—K1—O2^iv^	−51.2 (3)	C6—C1—S1—O1	−115.7 (5)
S1—N1—K1—O1^i^	−116.4 (2)	C2—C1—S1—O1	63.6 (6)
Cl1—N1—K1—O1^i^	132.5 (2)	C6—C1—S1—N1	131.0 (5)
S1—N1—K1—O3^iv^	−130.2 (6)	C2—C1—S1—N1	−49.7 (6)

Symmetry codes: (i) −*x*+1, −*y*+1, −*z*; (ii) *x*, *y*, *z*+1; (iii) *x*, −*y*+1/2, *z*+1/2; (iv) *x*, −*y*+1/2, *z*−1/2; (v) *x*, *y*, *z*−1.

### Hydrogen-bond geometry (Å, º)

**Table:**

*D*—H···*A*	*D*—H	H···*A*	*D*···*A*	*D*—H···*A*
O3—H31···N1^i^	0.85 (2)	2.06 (2)	2.901 (9)	173 (9)
O3—H32···Cl1^vi^	0.85 (2)	2.86 (5)	3.603 (6)	148 (9)

Symmetry codes: (i) −*x*+1, −*y*+1, −*z*; (vi) −*x*+1, *y*−1/2, −*z*−1/2.
